# Phenotypic variability in Meckel-Gruber syndrome

**DOI:** 10.1186/2046-2530-4-S1-P1

**Published:** 2015-07-13

**Authors:** S Darouich, I Bettaieb, M Khila, T Kitova, D Chelli, E Sfar, MB Channoufi, H Reziga, S Gaigi, A Masmoudi

**Affiliations:** 1 Embryo-Foetopathology Unit, Maternity and Neonatology Center, Salah Azaiez Institut, Tunis, Tunisia; 2 Immunohistocytology Department, Salah Azaiez Institut, Tunis, Tunisia; 3 Department 'C' of Gynecology and Obstetrics, Salah Azaiez Institut, Tunis, Tunisia; 4 Department of Anatomy, Histology and Embryology, Medical University of Plovdiv, Plovdiv, Bulgaria; 5 Department 'A' of Gynecology and Obstetrics, Salah Azaiez Institut, Tunis, Tunisia; 6 Department 'B' of Gynecology and Obstetrics, Salah Azaiez Institut, Tunis, Tunisia

## Objective

Meckel-Gruber syndrome (MGS) is a clinically and genetically heterogeneous disease with severe multisystem manifestations, associated with mutation in primary cilia-related genes. It is marked by a characteristic triad of major abnormalities: brain malformation (typically encephalocele), hepatorenal fibrocyctic changes and polydactyly. The aim of the study was to present a comprehensive analysis of autopsy findings in fetuses with MGS and to emphasise the phenotypic variability in this ciliopathy.

## Methods

We retrospectively examined the clinico-pathological findings in ten fetuses with MGS. The autopsies were performed after medical interruption of pregnancy due to fetal malformations.

## Results

All cases had bilateral and symmetrical enlargement of the kidneys with abdominal distension and lung hypoplasia. Nine fetuses had classical clinical triad. Polydactyly was absent in one case. Brain abnormalities consisted of encephalocele in nine cases (with molar tooth sign in two cases) and Dandy-Walker malformation in one case. Additional anomalies included microphthalmia (n = 1), aniridia (n = 2), cleft palate (n = 3), lobulated tongue (n = 3), micrognathia (n = 3), umbilical hernia (n = 1), genital abnormalities (n = 5), club foot (n = 5), cardiac septal defects (=1) and polysplenism (n = 2). Microscopically, the renal parenchyma demonstrated diffuse multicystic dysplasia. The liver showed fibrosis of the portal areas with variable degree of ductal proliferation and dilatation.

## Conclusion

Our data confirmed that MGS may demonstrate variation in phenotypic expression. However, occipital encephalocele or other central nervous system malformation and fibrocystic changes in in the kidney and liver are constant findings. Thus, meticulous autopsy is necessary to carefully assess all the various possible anomalies associated with MGS.

